# Three-Dimensional Speckle-Tracking Echocardiography-Derived Tricuspid Annular Dimensions and Right Atrial Strains in Healthy Adults—Is There a Relationship? (Insights from the MAGYAR-Healthy Study)

**DOI:** 10.3390/jcm12134240

**Published:** 2023-06-24

**Authors:** Attila Nemes, Árpád Kormányos, Zoltán Ruzsa, Alexandru Achim, Nóra Ambrus, Csaba Lengyel

**Affiliations:** Department of Medicine, Albert Szent-Györgyi Medical School, University of Szeged, Semmelweis Street 8, H-6725 Szeged, Hungary

**Keywords:** healthy, tricuspid annulus, right atrial, strain, three-dimensional, speckle-tracking, echocardiography

## Abstract

Introduction. The tricuspid valve and its annulus (TA) are thought to be integrally related to right atrial (RA) size and function. The present study aimed to assess associations between TA dimensions and RA strains, and quantitative features of its contractility were determined by 3DSTE in healthy adults. Methods. The study comprised 145 healthy volunteers with a mean age of 34.4 ± 12.5 years (73 males). Electrocardiographic, two-dimensional Doppler echocardiographic and 3DSTE parameters were in normal reference ranges in all subjects. Results. Enlarged TA areas, regardless of which phase of the cardiac cycle were measured, were not associated with the deterioration of peak RA strains in longitudinal (LS) and circumferential (CS) directions. Increased end-diastolic TA area was associated with reduced RA strain in the radial direction (RS). Dilation of end-diastolic and end-systolic TA areas was related to increased RA volumes. End-diastolic TA area was the smallest in case of increased peak global RA-RS, and other associations between increasing TA areas and peak global strains could not be detected. Peak global RA-CS and RA-LS were not related to TA areas. Increasing peak global RA-RS was not associated with peak global RA-LS and RA-CS, while increasing peak global RA-LS and RA-CS were not associated with peak global RA-RS. Increasing peak global RS did not show associations with RA volumes, V_min_ was the smallest in the case of highest peak global RA-CS and RA-LS. V_max_ increased with increasing peak global RA-LS. Conclusions. 3DSTE is suitable for simultaneous non-invasive determination of TA dimensions and RA volumes and strains using the same acquired 3D dataset, allowing physiologic studies. RA volumes are associated with end-diastolic and end-systolic TA areas. RA strains in radial direction (RS) show associations with end-diastolic TA area.

## 1. Introduction

Recently, there has been a growing interest in non-invasive testing of the right heart due to the introduction of new therapeutic procedures into daily use. We are increasingly using modern imaging methods that enable a much broader analysis than before, allowing clinical-physiologic studies for better understanding the right heart and its components, including the right ventricle (RV) and atrium (RA) and the tricuspid valve (TV) located between them. Moreover, studies conducted under healthy conditions can help to understand pathological variations [1].

During embryonic cardiac development, the heart is presented as a tube in early stages, which later transforms into a four-chambered organ. During embryogenesis, superior and inferior endocardial cushions become thickened areas, with development giving rise to TV, mitral valve, and cardiac septum by a fusion due to a subset of endothelial cells underwent epithelial-mesothelial transition. These facts highlight the close cooperation between the atria, ventricles, and atriventricular valves of the left and right hearts due to common (partial) development [2].

TV is an atrioventricular valve located on the right side, allowing blood flow from the RA to the RV, having a typical function of opening in diastole and closing in systole [3,4]. The TV has anterior, posterior, and septal leaflets, which attach to the ring called the tricuspid annulus (TA). The free edges of the leaflets are attached to the apices of the RV papillary muscles via inelastic fibrous chordae tendineae. The normal TA is non-planar and D-shaped with a larger C-shaped structure, corresponding to the RA/RV free wall and a straight segment corresponding to the septal leaflet/ventricular septum. Although TA was referred to as a fibrous structure, muscle bars were found in the TA itself. Therefore, TV/TA is thought to be integrally related to RA and RV size and function [3,4]. 

Although TA is difficult to define on surgical inspection, novel imaging techniques such as real-time three-dimensional (3D) echocardiography (RT3DE) and speckle-tracking echocardiography (3DSTE) can help with the accurate measurement of TA dimensions [5,6,7,8]. Moreover, using the same acquired 3D datasets during 3DSTE, even at the same time as the TA, volumetric and functional features of RA could be simultaneously determined in order to help us understanding the physiologic associations between TA and RA [8,9]. Therefore, the present study aimed to assess RA and TA dimensions and functional properties by 3DSTE and to compare their associations in healthy adults.

## 2. Patients and Methods

### 2.1. Study Population

The study comprised 145 healthy volunteers with a mean age of 34.4 ± 12.5 years (18–72 years, 73 males, weight: 70.2 ± 13.5 kg, height: 172.0 ± 9.6 cm, body surface area: 1.85 ± 0.21 m^2^, body mass index: 23.7 ± 3.7 kg/m^2^, systolic blood pressure: 123.2 ± 5.3 mm Hg, diastolic blood pressure: 78.3 ± 4.3 mm Hg). All cases were enrolled between 2011–2015 and all subjects underwent a complete examination, including physical examination, electrocardiography (ECG), two-dimensional Doppler echocardiography (2DE), and 3DSTE. Single observer 3DSTE assessment was performed in all cases (ÁK). A subject was considered healthy if he/she had no acute or chronic disorder, pathological state, or any abnormality affecting the results, and if there was no drug use, smoking, or obesity in his/her medical history. ECG, 2DE, and 3DSTE showed results in the normal reference ranges. The present study is part of the **M**otion **A**nalysis of the heart and **G**reat vessels b**Y** three-dimension **A**l speckle-t**R**acking echocardiography in **Healthy** subjects **(MAGYAR-Healthy) Study** aiming to investigate physiological relationships among 3DSTE-derived and other parameters in healthy adults (’Magyar’ means ’Hungarian’ in the Hungarian language) [8,9,10,11]. The study was conducted in accordance with the Declaration of Helsinki (as revised in 2013, and the updated versions). The Institutional and Regional Human Biomedical Research Committee of the University of Szeged, Hungary approved the study on a separate registration number (71/2011 and updated versions) and informed consent was given by all subjects.

### 2.2. Two-Dimensional Doppler Echocardiography

Toshiba Artida^TM^ echocardiographic tool (Toshiba Medical Systems, Tokyo, Japan) was applied for routine 2DE assessments using a 1–5 MHz PST-30BT phased-array transducer. Chamber quantifications were performed according to the recent guidelines [12]. Valvular regurgitations were quantified by visual assessments, while significant valvular stenoses were excluded by Doppler echocardiography. For determination of LV diastolic function, mitral inflow velocities and their ratio were measured and calculated. Representing systolic longitudinal motion of the TA, TA plane systolic excursion was also measured (TAPSE) [12,13,14].

### 2.3. Three-Dimensional Speckle-Tracking Echocardiography

After changing the transducer to a 3D capable PST-25SX matrix-array transducer attached to the same Toshiba Artida^TM^ equipment, the same protocol was used as described in previous MAGYAR-Healthy Study papers. Firstly, following optimalisation of image quality on gain and magnitude under ECG control, pyramid-shaped 3D echocardiographic datasets (called ‘echocloud’) were acquired from the apical window with the patient being on breathhold, the acquisition was focused on the RA. The echoclouds were digitally stored on hard drive for later analysis [15,16,17,18]. 

### 2.4. Quantification of 3DSTE-Derived RA Volumes and Strains

**Figure 1:**
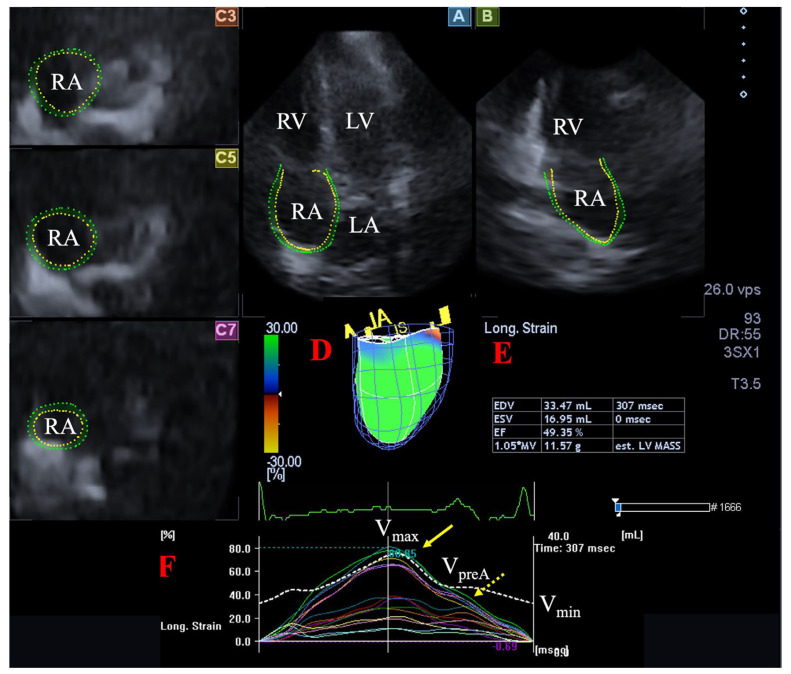
Assessment of the right atrium (RA) is presented extracted from a three-dimensional (3D) echocardiographic full-volume dataset: apical four-chamber view (**A**), apical two-chamber view (**B**), short-axis views at basal (**C3**), midatrial (**C5**), and superior (**C7**) RA levels. Virtual 3D cast of the RA (**D**), calculated RA volumes (**E**) and RA volume changes (dashed white line), and global and segmental RA (longitudinal) strain changes (white and coloured lines) over time (**F**) are also presented. Yellow arrow represents peak RA strains (dashed yellow arrow represents RA strains at atrial contraction, not examined). Abbreviations. LA = left atrium, LV = left ventricle, RA = right atrium, RV = right ventricle, V_max_ = end-systolic maximum RA volume, V_min_ = end-diastolic minimum RA volume, V_preA_ = preatrial contraction RA volume.

During offline analysis, the vendor-provided 3D Wall Motion Tracking software version 2.7 (Ultra Extend, Toshiba Medical Systems, Tokyo, Japan) was used. Automatically selected apical two-chamber (AP2CH) and four-chamber (AP4CH) views and 3 short-axis views at basal, midatrial and superior levels acquired at end-diastole were used for the presentation of the acquired 3D data, and data were displayed on 2D images. Then, a 3D RA model was created by using reference points on the RA endocardium in AP2CH and AP4CH views on the edges of the TA ring and the RA apex at end-diastole. Following these definitions, automatic sequential analysis (reconstruction) was started. Using the created 3D cast, the following RA volumes were calculated (Figure 1) [9,19]:

- Maximum RA volume, measured at end-systole, just before tricuspid valve opening (V_max_).
- RA volume before atrial contraction, measured at early-diastole at the time of P wave on ECG (V_preA_).
- Minimum RA volume measured at end-diastole, just before tricuspid valve closure (V_min_).

The following RA strains were determined regarding the RA reservoir function in end-systole (peak strains) [9,20]:

- Radial strain (RS) representing the thickening/thinning of a certain RA segment.
- Longitudinal strain (LS) representing the lengthening/shortening of a certain RA segment.
- Circumferential strain (CS) representing the widening/narrowing of a certain RA segment.

Global (assessing the whole RA), mean segmental (average of 16 segmental strains) and basal regional (average of 6 basal strains) peak RA strains were calculated.

### 2.5. Quantification of 3DSTE-Derived TA Dimensions

**Figure 2:**
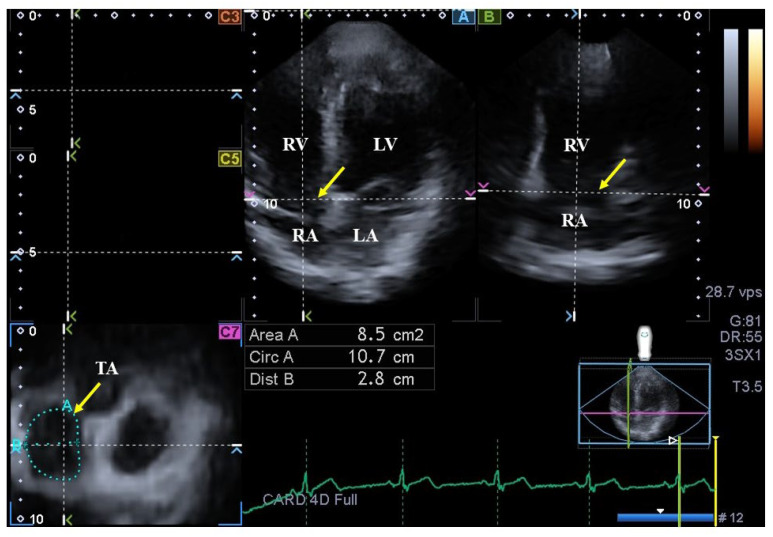
Assessment of the tricuspid annulus (TA) is presented extracted from a three-dimensional echocardiographic full-volume dataset: apical four-chamber view (**A**); apical two-chamber view (**B**); and a cross-sectional view at the level of the TA optimized in apical four- and two-chamber views (**C7**); short-axis views at basal (**C3**), midatrial (**C5**). The yellow arrow represents the tricuspid annular plane. Abbreviations: LA = left atrium, LV = left ventricle, TA = tricuspid annulus, RA = right atrium, RV = right ventricle, Area = TA area, Circ = TA perimeter, Dist = TA diameter.

On AP2CH and AP4CH views, using the optimal lateral and septal TA endpoints on C7 short-axis view, TA area (TAA) was measured by planimetry in end-systole (TAA-S) and end-diastole (TAA-D)(Figure 2), and TA fractional area change (TAFAC) was then calculated using the following equation [5,6,7,8,21]: TAFAC = ([TAA-D – TAA-S]/TAA-D) × 100.

### 2.6. Statistical Analysis

Depending on variables being continuous or dichotomous, data were presented in mean ± standard deviation (SD) format or frequency/percentage format, as appropriate. Statistical significance was considered in case of *p* < 0.05. For continuous variables, Student *t* test with Welch correction and one-way analysis of variance (ANOVA) test with Bonferroni correction were used, as appropriate. For categorical variables, Fisher’s exact test was used. Pearson’s correlation coefficient was calculated for correlations. SPSS software version 22 (SPSS Inc., Chicago, IL, USA) was applied for statistical calculations.

## 3. Results

### 3.1. Two-Dimensional Doppler Echocardiography

2D echocardiography-derived variables including left atrial diameter measured in parasternal long-axis view (37.3 ± 3.6 mm), LV end-diastolic, and end-systolic diameters (48.1 ± 3.5 mm and 32.1 ± 3.0 mm, respectively), and volumes (105.6 ± 22.9 mL and 37.6 ± 9.4 mL, respectively), interventricular septum (9.2 ± 1.3 mm), LV posterior wall (9.3 ± 1.5 mm) and LV ejection fraction (64.8 ± 3.9%) were all within the normal range. Early and late mitral inflow velocities and their ratio proved to be 80.1 ± 15.8 cm/s and 59.3 ± 14.2 cm/s, respectively. None of the healthy subjects showed valvular regurgitations or had significant valvular stenosis on any valves. 

### 3.2. Classification of Subjects

The peak global RA-RS, RA-CS, RA-LS, TAA-D and TAA-S proved to be −13.61 ± 8.13%, 19.52 ± 13.72%, 34.78 ± 14.04%, 7.31 ± 1.60 cm^2^ and 5.36 ± 1.44 cm^2^, respectively. Using the pool of healthy subjects, 3 groups were created according to the mean ± SD of 3DSTE-derived peak global RA-RS, RA-CS, RA-LS, TAA-D and TAA-S: estimated mean ± SD served as the lower (-5.48%, 5.80%, 20.74%, 5.71 cm^2^ and 3.92 cm^2^, respectively) and upper (−21.74%, 33.24%, 48.82%, 8.91 cm^2^ and 6.80 cm^2^, respectively) values. 

### 3.3. Dilation of TA and RA Volumes and Function

**Figure 3:**
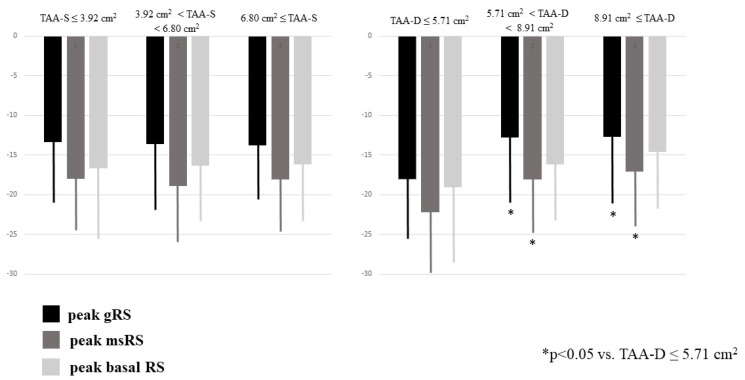
Peak global, mean segmental and basal right atrial radial strains in healthy subjects having different sized end-systolic and end-diastolic tricuspid annular areas as assessed by three-dimensional speckle-tracking echocardiography.

Dilation of TAA-S was associated with dilation of TAA-D and reduction of TAFAC. Dilation of TAA-D was associated with dilation of TAA-S and preservation of TAFAC. Enlarged TA areas, regardless in which phase of the cardiac cycle they were measured, were not associated with the deterioration of TAPSE or peak RA-LSs and RA-LCs. Dilation of TAA-D and TAA-S was related to increased RA volumes (Table 1). Increased TAA-D was associated with reduced RA-RSs (Table 1, Figure 3).

### 3.4. Increasing RA Strains and TA Dimensions and Function

**Figure 4:**
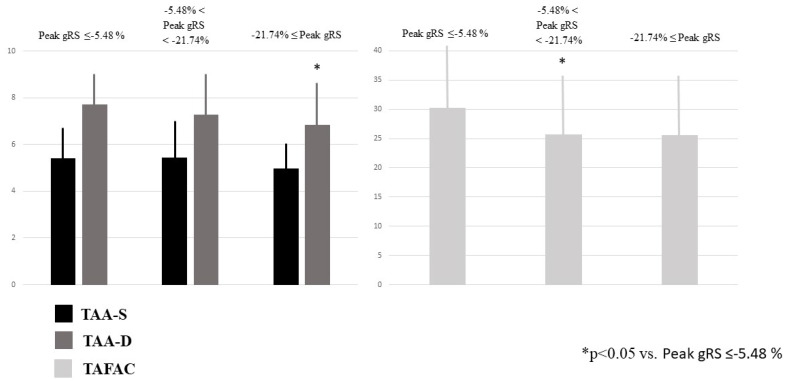
End-systolic and end-diastolic tricuspid annular areas and tricuspid annular fractional area change in healthy subjects having varying degrees of peak global right atrial radial strains as assessed by three-dimensional speckle-tracking echocardiography.

TAA-D was the smallest in case of increased peak global RA-RS, and other associations between increasing TA areas and peak global strains could not be detected. TAFAC was reduced in case of increased peak global RA-RS (Table 2, Figure 4). Peak global RA-CS and RA-LS were not related to TA areas. None of the peak global RA-strains were related to TAPSE. Increasing peak global RA-RS was not associated with peak global RA-LS and RA-CS, while increasing peak global RA-LS and RA-CS were not associated with peak global RA-RS. Increasing peak global RA-RS did not show associations with RA volumes, and V_min_ was the smallest in case of the highest peak global RA-CS and RA-LS. V_max_ increased with increasing peak global RA-LS (Table 2).

### 3.5. Tricuspid Annular Plane Systolic Excursion

TAPSE did not show any significance between the subgroups examined (Table 1 and Table 2).

### 3.6. Correlations

No significant correlations were detected between any RA and TA parameters. 

## 4. Discussion

In the past decade, new therapeutic procedures have appeared, with the help of which it has become possible to treat previously untreatable or barely treatable cardiovascular conditions. These include pathological conditions affecting the right heart, such as congenital and/or valvular heart diseases or cardiomyopathies. Non-invasive imaging methods used in routine allowed only limited visualization (such as TA and RA), but modern procedures have made it easy to examine the right heart [1]. 3DSTE is an optimal imaging technique for such analysis due to its non-invasive nature and easy-to-learn/easy-to-use ability [15,16,17,18], which allows simultaneous evaluation of 2D-projected measurement of TA dimensions and 3D cast-based analysis of RA volumes and strains at the same time using the same acquired 3D echocardiographic datasets [8,9]. Moreover, 3DSTE-derived normal reference values of these parameters are also determined [19,20,21]. To the best of the authors’ knowledge, this is the first clinical-physiologic study in which relationships between 3DSTE-derived RA strains and TA dimensions were examined in healthy adult subjects without functional tricuspid regurgitation (FTR). 

The main finding of the present study is that the dilation of TAA-D is associated with increased RA volumes and the reduction of peak RA-RSs. RA-CSs and RA-LSs did not show any relationship with TA dimensions. On the other hand, TAA-D was smallest in the presence of highest peak global RA-RS representing systolic function of the RA. Increasing TAA-S was associated with increasing RA volumes, but did not with peak RA strains. Changes in RA strains in circumferential and longitudinal directions were not associated with TA dimensions. Moreover, TAPSE, longitudinal movement of TA did not show any associations with RA strains and TA dimensions. 

These results should be evaluated in light of understanding of how closely RA and TA develop together during embryogenesis [2]. Summing up these results, it can be said that RA volumes have a significant role in the determination of TA dimensions in healthy adult subjects without FTR [22]. Moreover, radial RA strains are also associated with TA size, highlighting the significance of muscle bands not only in RA but also inside the TA as well in regulating TA dimensions and functional properties [4]. However, transmural fiber angle distribution is heterogeneous throughout different regions of the atria, which could partially explain why associations with RA-LS and RA-CS could not be detected [23]. In a recent study, the Frank–Starling mechanism for RA was examined and demonstrated that increasing RA volumes do not cause a significant increase in peak RA strains representing reservoir function in systole [11]. This finding was extended in this study, demonstrating that increased RA contractility in longitudinal direction represented by peak RA-LS is associated with larger V_max_ and lower V_min_. 

These findings could help us understanding the physiologic relationship between RA and TA in healthy circumstances. However, further studies are needed to confirm the presented results in larger patient populations. Furthermore, the role of the presented factors in the designation of FTR should also be further investigated, even in certain pathological states [22]. 

### Limitation Section

Several important limitations have arisen during our assessments:

- The issue of image quality of echocardiographic analysis is still a pivotal point, for 3DSTE it is still worse than that of 2D echocardiography, which should be taken into account when interpreting the results [15,16,17,18]. Nevertheless, considering both the advantages and disadvantages, the clinical role of 3DSTE is unquestionable.
- As mentioned above, with 3D echocardiography, the 2D-projected D shape of TA could be analysed, not its real 3D shape [5,8].
- The present study did not propose further validation of 3DSTE-derived TA and RA quantifications.
- STE-derived analysis of TA functional properties were not an aim of the study.
- FTR was excluded by visual assessment, and a more advanced quantification method was not performed during the present study.

## 5. Conclusions

3DSTE is suitable for simultaneous non-invasive determination of TA dimensions and RA volumes and strains using the same acquired 3D dataset allowing physiologic studies. RA volumes are associated with end-diastolic and end-systolic TA areas. RA strains in radial direction (RS) show associations with end-diastolic TA area.

## Figures and Tables

**Table 1:** Peak global and regional right atrial strains in different end-diastolic tricuspid annular subgroups.

	TAA-S ≤ 3.92 cm^2^ (*n* = 23)	3.92 cm^2^ < TAA-S < 6.80 cm^2^ (*n* = 105)	6.80 cm^2^ ≤ TAA-S (*n* = 17)	TAA-D ≤ 5.71 cm^2^ (*n* = 23)	5.71 cm^2^ < TAA-D < 8.91 cm^2^ (*n* = 98)	8.91 cm^2^ ≤ TAA-D (*n* = 24)
TAA-S (mm^2^)	3.47 ± 0.35	5.34 ± 0.78 *	8.08 ± 1.27 *†	3.74 ± 0.57	5.24 ± 0.92 **	7.33 ± 1.54 **††
TAA-D (mm^2^)	5.47 ± 0.95	7.31 ± 1.16 *	9.79 ± 1.39 *†	5.04 ± 0.51	7.19 ± 0.85 **	9.81 ± 1.04 **††
TAFAC (%)	35.3 ± 10.1	26.1 ± 9.8 *	17.1 ± 6.1 *†	25.5 ± 10.2	26.9 ± 10.7	26.2 ± 10.5
TAPSE (mm)	23.2 ± 2.9	24.1 ± 2.9	23.3 ± 2.9	24.5 ± 3.6	23.8 ± 2.8	23.9 ± 3.0
peak gRS (%)	−13.4 ± 7.6	−13.6 ± 8.5	−13.8 ± 6.6	−18.0 ± 7.6	−12.8 ± 8.0 **	−12.7 ± 8.0 **
peak msRS (%)	−18.0 ± 6.5	−18.9 ± 7.2	−18.1 ± 6.4	−22.2 ± 7.6	−18.1 ± 6.6 **	−17.1 ± 7.2 **
peak basal RS (%)	−16.7 ± 8.8	−16.3 ± 7.3	−16.2 ± 7.0	−19.1 ± 9.0	−16.2 ± 7.0	−14.6 ± 7.4
peak gCS (%)	19.5 ± 12.4	19.5 ± 13.2	19.8 ± 17.8	18.9 ± 13.5	19.2 ± 13.4	21.6 ± 15.0
peak msCS (%)	25.1 ± 12.0	25.8 ± 12.9	25.2 ± 18.4	25.6 ± 13.8	25.2 ± 13.0	27.2 ± 15.4
peak basal CS (%)	27.0 ± 12.3	26.0 ± 11.5	24.4 ± 11.7	28.5 ± 16.3	25.4 ± 10.8	26.4 ± 9.7
peak gLS (%)	32.0 ± 17.7	35.4 ± 13.4	34.3 ± 12.2	35.4 ± 18.6	33.9 ± 13.1	37.9 ± 12.4
peak msLS (%)	36.2 ± 16.8	39.4 ± 13.0	38.2 ± 11.0	39.6 ± 18.2	38.1 ± 12.5	40.9 ± 11.9
peak basal LS (%)	34.8 ± 20.1	44.9 ± 19.8	40.9 ± 16.8	46.1 ± 28.3	41.7 ± 17.2	44.9 ± 19.7
V_max_ (mL)	34.0 ± 8.6	50.0 ± 13.1 *	61.3 ± 17.8 *†	36.4 ± 9.3	48.8 ± 13.6 **	59.8 ± 16.4 **††
V_preA_ (mL)	25.0 ± 7.0	36.3 ± 8.7 *	46.2 ± 14.9 *†	27.3 ± 7.7	35.8 ± 9.5 **	43.1 ± 13.5 **††
V_min_ (mL)	18.8 ± 6.7	28.3 ± 8.4 *	35.6 ± 11.3 *†	20.5 ± 6.5	27.9 ± 8.9 **	33.6 ± 10.6 **††

Abbreviations. g = global, ms = mean segmental, RS = radial strain, CS = circumferential strain, LS = global longitudinal strain, TAA-S = end-systolic tricuspid annular area, TAA-D = end-diastolic tricuspid annular area, TAFAC = tricuspid annular fractional area change, TAPSE = tricuspid annular plane systolic excursion, V_max_ = end-systolic maximum right atrial volume, V_preA_ = diastolic pre-atrial contraction right atrial volume, V_min_ = end-diastolic minimum right atrial volume. * *p* < 0.05 vs. TAA-S ≤ 3.92 cm^2^; † *p* < 0.05 vs. 3.92 cm^2^ < TAA-S < 6.80 cm^2^; ** *p* < 0.05 vs. TAA-D ≤ 5.71 cm^2^; †† *p* < 0.05 vs. 5.71 cm^2^ < TAA-D < 8.91 cm^2^.

**Table 2:** Global peak right atrial strains and tricuspid annular parameters in different right atrial strain subgroups.

	Peak gRS ≤ −5.48% (*n* = 27)	5.48% < Peak gRS < −21.74% (*n* = 95)	−21.74% ≤ Peak gRS (*n* = 23)	Peak gCS ≤ 5.80% (*n* = 19)	5.80% < Peak gCS < 33.24% (*n* = 111)	33.24% ≤ Peak gCS (*n* = 15)	Peak gLS ≤ 20.74% (*n* = 24)	20.74% < Peak gLS < 48.82% (*n* = 102)	48.82% ≤ Peak gLS (*n* = 19)
TAA-S (mm^2^)	5.40 ± 1.32	5.44 ± 1.55	4.98 ± 1.00	5.08 ± 1.54	5.46 ± 1.46	5.04 ± 1.05	4.93 ± 1.38	5.51 ± 1.48	5.15 ± 1.29
TAA-D (mm^2^)	7.72 ± 1.29	7.29 ± 1.66	6.83 ± 1.52 *	6.73 ± 1.68	7.46 ± 1.46	6.91 ± 1.35	6.85 ± 1.70	7.43 ± 1.58	7.19 ± 1.58
TAFAC (%)	30.3 ± 10.6	25.7 ± 10.4 *	25.6 ± 10.3	24.7 ± 10.6	26.8 ± 10.6	26.6 ± 10.6	27.8 ± 10.5	26.0 ± 10.3	27.7 ± 12.4
TAPSE (mm)	24.2 ± 2.8	23.8 ± 2.9	23.9 ± 3.3	24.0 ± 2.9	24.0 ± 2.9	22.6 ± 2.5	24.7 ± 2.7	23.7 ± 3.0	24.1 ± 2.8
Peak gRS (%)	−2.8 ± 2.1	−13.3 ± 4.7 *	−26.0 ± 4.5 *†	−14.2 ± 10.4	−13.6 ± 7.8	−13.1 ± 7.4	−12.7 ± 6.1	−13.9 ± 8.4	−12.7 ± 8.3
Peak gCS (%)	20.3 ± 14.2	18.9 ± 13.2	21.9 ± 15.2	2.5 ± 2.3	18.4 ± 7.2 **	48.6 ± 14.6 **††	13.2 ± 10.8	18.6 ± 12.3 ***	32.9 ± 15.3 ***†††
Peak gLS (%)	34.7 ± 14.7	33.5 ± 13.5	39.0 ± 14.3	28.1 ± 12.2	34.0 ± 12.2 **	48.9 ± 18.6 **††	15.9 ± 4.2	34.2 ± 7.7 ***	59.9 ± 10.1 ***†††
V_max_ (mL)	48.0 ± 16.4	48.2 ± 14.1	51.9 ± 16.3	45.9 ± 16.2	48.6 ± 14.6	54.5 ± 15.7	44.7 ± 12.5	48.6 ± 15.2 ***	54.8 ± 15.3 ***
V_preA_ (mL)	35.2 ± 12.0	35.6 ± 10.4	36.6 ± 11.6	37.7 ± 13.5	35.8 ± 10.4	33.6 ± 11.2	37.6 ± 11.2	35.8 ± 10.9	33.0 ± 10.1
V_min_ (mL)	27.1 ± 10.3	27.9 ± 9.4	27.6 ± 9.8	32.3 ± 11.0	27.7 ± 9.3	22.9 ± 8.1 ††	32.0 ± 10.7	27.7 ± 9.4	23.1 ± 7.2 ***†††

Abbreviations. g = global, RS = radial strain, CS = circumferential strain, LS = longitudinal strain, TAA-S = end-systolic tricuspid annular area, TAA-D = end-diastolic tricuspid annular area, TAFAC = tricuspid annular fractional area change, TAPSE = tricuspid annular plane systolic excursion, V_max_ = end-systolic maximum right atrial volume, V_preA_ = diastolic pre-atrial contraction right atrial volume, V_min_ = end-diastolic minimum right atrial volume. * *p* < 0.05 vs. Peak gRS ≤ −5.48%; † *p* < 0.05 vs. −5.48% < Peak gRS < −21.74%; ** *p* < 0.05 vs. Peak gCS ≤ 5.80%; †† *p* < 0.05 vs. 5.80% < Peak gCS < 33.24%; *** *p* < 0.05 vs. Peak gLS ≤ 20.74%; ††† *p* < 0.05 vs. 20.74% < Peak gLS < 48.82%.

## Data Availability

All data are available.
